# Urocortin stimulates the ERK1/2 signaling pathway and the proliferation of HeLa cells via CRF receptor 1

**DOI:** 10.1002/2211-5463.13602

**Published:** 2023-04-02

**Authors:** Bálint Balogh, Mónika Vecsernyés, Alexandra Stayer‐Harci, Gergely Berta, Oktávia Tarjányi, György Sétáló

**Affiliations:** ^1^ Department of Medical Biology and Central Electron Microscope Laboratory, Medical School University of Pécs Hungary; ^2^ Signal Transduction Research Group János Szentágothai Research Centre Pécs Hungary

**Keywords:** cell proliferation, E2F‐1, ERK1/2, MEK, HeLa, urocortin

## Abstract

Corticotropin‐releasing factor (CRF) stimulates adrenocorticotropic hormone (ACTH) secretion from the pituitary gland and is an essential regulator of the hypothalamic–pituitary–adrenocortical axis. Isoforms of CRF receptor are known to mediate the effects of urocortin stress ligands on the regulation of stress responses, anxiety, and feeding behavior; however, urocortin stress ligands also influence cell proliferation. In view of the tumor‐promoting capacity of prolonged stress, here we investigated (a) the effect of urocortin on cell proliferative signaling via extracellular signal‐regulated kinase 1/2, (b) the expression and cellular distribution of the specific CRF receptor isoforms, and (c) the intracellular localization of phosphorylated ERK1/2 in HeLa cells. Stimulation of cell proliferation was observed in the presence of 10 nm urocortin. Our data also suggest that MAP kinase MEK, the transcription factors E2F‐1 and p53, and PKB/Akt are involved in this process. These findings may have therapeutic relevance for the targeted treatment of various malignancies.

## Graphical abstract

We investigated the signaling and biological effects of human urocortin 1 (hUCN1) in HeLa cells. hUCN1 treatment stimulates ERK1/2 MAPK phosphorylation, which is initiated by CRF1 and then mediated via MEK. The S‐phase gene‐activating transcription factor E2F‐1 is also induced, leading eventually to increased cell proliferation, as demonstrated by MTT assay.

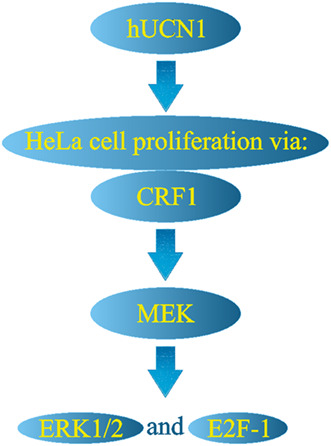

**Abbreviations**
A2b: Astressin 2b, a CRF2‐specific inhibitor
ACTH: adrenocorticotropic hormone
Ant: antalarmin, a CRF1‐specific inhibitor
ATCC: American Type Culture Collection
cAMP: cyclic adenosine monophosphate
CBP: CRF‐binding protein
Class B1 GPCR: class B1 G protein‐coupled receptors
CNS: central nervous system
CRF: corticotropin‐releasing factor
CRF1 and CRF2: corticotropin‐releasing factor receptors 1 and 2
DMEM: Dulbecco's Modified Eagle's Medium
DMSO: dimethyl sulfoxide
E2F‐1: S‐phase‐gene‐inducing transcription factor family member
EDTA: ethylene diamine tetraacetic acid
EGTA: ethylene glycol tetraacetic acid
ERK1/2: extracellular signal‐regulated kinase 1 and 2
G protein: guanine nucleotide‐binding protein
HEK 293: human embryonic kidney cells
HeLa: Henrietta Lacks, human cervical adenocarcinoma cell line
HL‐1: cardiac muscle cell line
HRP: horseradish peroxidase
hUCN1: human urocortin 1
MCF7: Michigan Cancer Foundation 7, human breast adenocarcinoma cell line
MEK: mitogen‐activated protein kinase/ERK kinase
p53: a tumor suppressor protein, its molecular weight is 53 kDa
PBS: phosphate‐buffered saline
pERK1 and pERK2: phosphorylated ERK1 and 2
PI3K: phosphatidylinositol 3 Kinase
PKA: protein kinase A
PKB/Akt: protein kinase B, also known as Akt protein
PKC: protein kinase C
PMSF: phenyl methane sulfonyl fluoride
PVDF: polyvinylidene difluoride
Raf‐1: a protein kinase isolated from rapidly proliferating fibrosarcoma
RM‐1: mouse prostate cancer cell line
SDS: sodium dodecyl sulfate
TBS: Tris‐buffered saline
THP‐1: human monocyte‐derived from acute monocytic leukemia
U0126: a chemical inhibitor of MEK
UCN: urocortin
Y79: human retinoblastoma cell line

Corticotropin‐releasing factor (CRF) is a 41‐amino acid‐long polypeptide that stimulates adrenocorticotropic hormone (ACTH) secretion from the pituitary gland [1] and is an essential regulator of the hypothalamic–pituitary–adrenocortical axis [2]. In addition, CRF is a vital regulator of stress reactions and immune responses, and has a positive effect on the cardiovascular system [3]. CRF and CRF‐like peptides, such as urotensin, sauvagine, and urocortins (UCNs) act on class B1 of G protein‐coupled receptors (GPCRs) that possess seven‐transmembrane domains and are known as CRF1 and CRF2 [4, 5]. In humans, members of the GPCRs are encoded by 15 genes and they generally convey autocrine or paracrine effects [5]. CRF1 is expressed in the central nervous system (CNS) and the anterior pituitary gland, while CRF2 has three splice variants, CRF2‐α, β and γ, the latter being only expressed in the human brain [4] CRF2‐α is expressed throughout the CNS, while CRF2‐β is preferentially expressed in peripheral tissues, such as the heart, skin, and skeletal muscle [6]. CRF receptors are also present in various human cancers, such as melanomas, small‐cell lung cancer, and neuroblastoma [3].

In 1995 a new mammalian member of the CRF family was described by Vaughan *et al*. [7]. The peptide named urocortin is related to CRF and urotensin. Three main types of urocortin are known, urocortin1 (UCN1), urocortin2 (UCN2, also known as stresscopin‐related peptide), and urocortin3 (UCN3, or stresscopin). These neuropeptides are synthesized in the central nervous system, they have varying distribution patterns and are also detectable in numerous peripheral tissues [8]. The *in vitro* binding affinities of UCN1 to CRF1 and CRF2 are essentially equal and it is up to 10‐fold higher to CRF1 than the binding of CRF itself to this receptor isoform [7, 9]. In addition, CRF, UCN2, and UCN3 all bind with basically equal affinities to CRF2 the latter two ligands also being selective for this receptor isoform [9, 10]. *In vitro* binding affinity, however, cannot be regarded as a direct indicator of *in vivo* efficacy as also demonstrated, for example, by Hasdemir *et al*. [11]. In addition, CRF‐binding protein (CBP) is an important regulator of CRF and UCN1 activity. It is commonly regarded as an endogenous buffer for CRF and UCN, regulating ligand availability for CRF receptors [8]. Members of the GPCRs' families can bind to various heterotrimeric G‐proteins leading to rather promiscuous signaling patterns [5]. Ligand binding of CRF1 and CRF2 stimulates adenylate cyclase activity, which increases cAMP production that in turn activates Protein Kinase A (PKA) [12, 13]. Numerous studies suggest that members of the CRF family exert their effects through Gsα leading to increased cAMP production, but other second messengers and signaling components, such as MAPK pathways are also implicated [14].

In this study, we investigated the effects of human urocortin1 (hUCN1) in culture on the signaling of HeLa cells with special emphasis on the expression and cellular distribution of the CRF receptors, the activating phosphorylation, and intracellular localization of the extracellular signal‐regulated kinases (ERK1 and 2). The involvement of Protein Kinase B (PKB)/Akt, and that of p53 was also examined, together with possible biological responses of the cells, like changes in proliferation. In comparison to our previous observations in MCF7 human breast cancer cells [15] we could detect both similarities and important differences in the signaling patterns induced by hUCN1 in HeLa cells, also accompanied by important differences in the elicited biological responses.

## Materials and methods

### Cell culture

HeLa cervical adenocarcinoma cells (purchased from ATCC and generously gifted by Professor József Szeberényi) were regularly maintained under standard circumstances (37 °C, 5% CO_2_ in a humidified atmosphere) in Dulbecco's Modified Eagle's Medium (DMEM) complemented with 10% heat‐inactivated fetal bovine serum for 24 h to achieve sufficient adhesion as described earlier in an article published by our research group [15]. The cells were cultured on plastic Petri dishes or plastic Thermanox (Nalgene Nunc International, Rochester, NY, USA) coverslips. The media were routinely replaced by 0.5% heat‐inactivated horse serum‐containing DMEM 1 day before the applied treatments to silence serum‐stimulated mitogenic signaling.

### Reagents

hUCN1 was dissolved in 10% acetic acid according to the instructions of the manufacturer. The MEK inhibitor U0126 and the CRF1‐specific inhibitor Antalarmin were dissolved in DMSO, while the CRF2‐specific inhibitor Astressin 2b was in distilled water. We also confirmed that the vehicle of the applied agents had no effect on the examined parameters of the experiments, as published in a former article [15]. All chemicals were purchased from Sigma‐Aldrich (a member of Merck Group, Budapest, Hungary) unless otherwise stated.

### Western blotting

HeLa cells were treated with 10 nm final concentration of hUCN1 for the indicated times or were left untreated as control. The applied inhibitors were added to the culturing media 30 min before the start of hUCN1 treatments at the following final concentrations: U0126 at 20 μm, Antalarmin at 100 nm, Astressin 2b at 100 nm. Upon completion of treatments, the cultures were harvested into ice‐cold lysis buffer (50 mm Tris‐base, pH 7.4, 150 mm NaCl, 10% glycerol, 1 mm EGTA, 1 mm Na‐orthovanadate, 5 μm ZnCl_2_, 100 mm NaF, 10 μg·mL^−1^ aprotinin, 1 μg·mL^−1^ leupeptin, 1 mm PMSF, 1% Triton X‐100) and were frozen for storage. Subsequent processing included thawing and homogenization by vortexing for 20 s, then the samples were centrifuged at 13 500 × ***g*** and at 4 °C for 20 min and the protein content of the supernatant was measured using a Protein Assay Dye Reagent Concentrate (Bio‐Rad, Hercules, CA, USA). Equal amounts of proteins (30 μg) were mixed with 4× Laemmli buffer (25 mL 1 m Tris–HCl, pH 6.8, 40 mL glycerol, 8 g SDS, 10 mL 100 mm EDTA, 10 mL 100 mm EGTA, and 1 mL 1% bromophenol blue brought up to 100 mL with distilled water) and boiled briefly to denature proteins. Protein samples were loaded onto 10% polyacrylamide gels and separated according to molecular weight. The proteins were electro‐blotted onto PVDF membranes (Hybond‐P, GE Healthcare, Buckinghamshire, UK) by using a Transfer Blot Turbo System (Bio‐Rad). After the transfer of the proteins, we blocked the membranes in 5% nonfat dry milk (or in 3% bovine serum albumin in the case of phospho‐Akt detection) dissolved in TBS‐Tween (10 mm Tris‐base, 150 mm NaCl, 0.2% Tween‐20, pH 8.0). The primary antibodies were also diluted in the blocking solution (anti‐CRF1, Fine Biotech, PRC; anti‐CRF2, Sigma‐Aldrich; phospho‐ERK1/2, ERK1/2, pAKT, Akt, E2F‐1, and beta‐actin, Cell Signaling Technology, Beverly, MA, USA or p53, Santa Cruz Biotechnology, Santa Cruz, CA, USA) 1 : 1000 and incubated overnight. Methodical controls prepared by the omission of the primary antibodies or the use of nonimmune sera of the appropriate species resulted in no signal production as determined in pilot experiments (latter data not shown). To remove unbound antibodies, we washed the membranes five times in TBS‐Tween, then incubation followed with a horseradish‐peroxidase (HRP)‐conjugated anti‐rabbit or anti‐mouse secondary antibody (Cell Signaling Technology) dissolved 1 : 5000 in the blocking solution. Five washes in TBS‐Tween removed the unbound antibodies. Enhanced chemiluminescence detection of the target proteins followed (Immobilon Western, Millipore Corporation, Billerica, MA, USA) using a G‐box gel documentation system (Syngene, Cambridge, UK). Then, densitometry was performed using the imagej software (National Institutes of Health, Bethesda, MD, USA).

### Data presentation of western blots

As described in detail in an article published earlier by our research group [15], all experiments were repeated at least three times. Data were normalized to the mean of the corresponding control (untreated) group. Significance of differences was determined by graphpad prism 9.5.0 (GraphPad Software, Boston, MA, USA) using one‐way (for proteins possessing one isoform) or two‐way (for proteins exhibiting two isoforms) ANOVA testing applying the appropriate corrections, as also indicated in the figure legends. *P* values ˂ 0.05 were considered significant. The relevant intensity differences are marked in the graphs and their corresponding *P* values are also written into the figure legends. Presented images are representative of series that produced similar results. Densitometric values are displayed by box + whiskers plots indicating median (central horizontal line), min/max values, and mean (with ‘+’ within the box).

### Confocal microscopy

HeLa cells were treated in the presence of 10 nm hUCN1 for the indicated times or left untreated as control. As described in our earlier publication [15], treatments were stopped by quickly rinsing in 37 °C PBS (1.37 mm NaCl, 0.27 mm KCl, 0.43 mm Na_2_HPO_4_·7H_2_O, 0.14 mm KH_2_PO_4_, pH 7.4) and then fixing in 4% paraformaldehyde dissolved in PBS (pH 7.4) for 1 h followed at room temperature. Three changes of PBS and three changes of TBS (50 mm Tris–HCl, pH 7.4, 150 mm NaCl) removed the excess fixative. Then, the samples were incubated in 5% nonfat dry milk dissolved in TBS‐Triton (50 mm Tris–HCl, pH 7.4, 150 mm NaCl, 0.1% Triton X‐100) at 4 °C for 1 h under gentle rocking to block nonspecific binding sites. Commercially available anti‐CRF1 (Fine Biotech, Wuhan, China) and anti‐CRF2 (Sigma‐Aldrich) primary antibodies were diluted 1 : 100. The dilution was 1 : 300 in the case of phospho‐ERK1/2 (Cell Signaling Technology). The diluent was always the corresponding blocking solution and incubation with the primary antibodies lasted overnight at 4 °C under gentle rocking. Unbound antibodies were removed by five washes in TBS. Cy3‐conjugated anti‐rabbit secondary antibody (Jackson Immuno Research, Cambridgeshire, UK) dissolved in the blocking solution was pipetted onto the samples at 4 °C for an hour at a dilution of 1 : 300 for CRF1 and 2 detections and at 1 : 600 to detect phospho‐ERK1/2. The omission of the primary antibody served as methodical control resulting in no detectable signal at the applied microscope settings.

### Data presentation of confocal micrographs

An Olympus FV‐1000 laser scanning system was used for confocal imaging, with a 40×, long distance, combined phase contrast‐ and fluorescence objective (NA:0.75) to capture single optical sections. The confocal aperture was set at 130 μm, excitation of the Cy3 dye conjugated to the secondary antibody happened at 543 nm, and the emission wavelength was 567 nm. Signal integration type was line Kalman. The micrographs shown were taken from series with similar results. All experiments have been repeated at least three times.

### MTT assay

Ninety‐six‐well plates were used to culture HeLa cervical adenocarcinoma cells (as described above) and then treated with 10 nm hUCN1 alone, or also with the CRF1 inhibitor Antalarmin or the CRF2 inhibitor Astressin 2b for 24 h. At the end of the treatments, we added 10 μL of Thiazolyl Blue Tetrazolium Bromide (Merck) dye solution (5 mg·mL^−1^) dissolved in sterile water to the cells' medium (100 μL per well). Incubation in the cell culture thermostat followed for an additional 4 h. Then, the supernatant was removed and the accumulated formazan product—the amount of which is proportional to the cells' metabolic activity—was eluted by adding a 1 : 1 mixture of 100 μL DMSO‐ethanol. The absorbance was determined at 595 nm using an ELISA microplate reader (FLUOstar OPTIMA, BMG Labtech, Ortenberg, Germany).

### Data presentation of MTT assay

One‐way ANOVA testing applying Tukey's corrections was used to determine the significance of differences. All experiments were repeated at least three times with samples prepared in triplicates. *P* values ˂ 0.05 were considered significant, which is also indicated in the figure legends. The results are the mean + SD for experiments performed independently.

## Results

### CRF receptor expression in HeLa cells

**Fig. 1:**
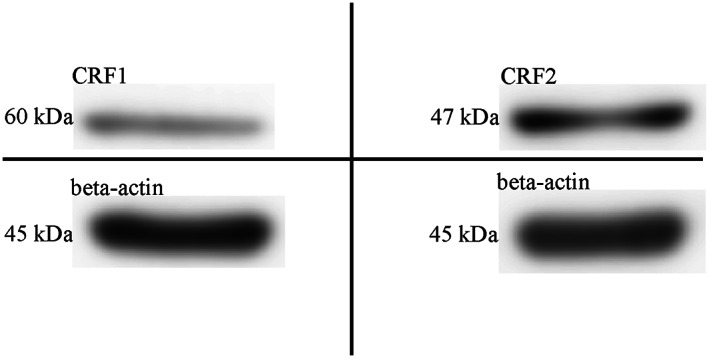
CRF1 and CRF2 expression in HeLa cells by western blotting. Detection of CRF1 (60 kDa, upper left quadrant) and CRF2 (47 kDa, upper right quadrant) expression using isoform‐specific antisera. The beta‐actin signal served as loading control (lower two quadrants).

We examined the expression of CRF receptors in HeLa cells by western blotting using commercially available antibodies that specifically recognize CRF1 and 2 subtypes. As shown in Fig. 1, we could detect both CRF1‐ and CRF2‐specific immunoreactivities at the expected positions on western blots (Fig. 1).

### Localization of CRF1 and CRF2 in HeLa cells by confocal microscopy

**Fig. 2:**
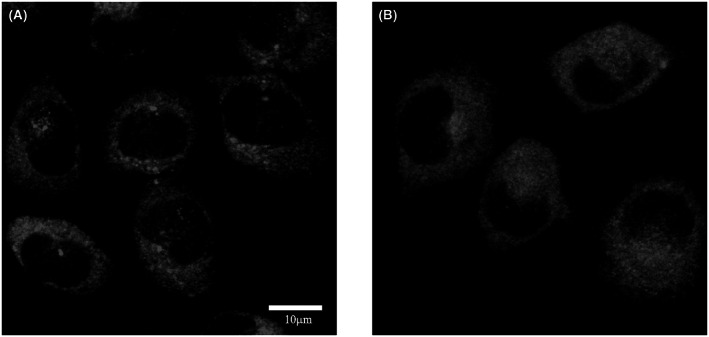
Intracellular localization of CRF receptors in HeLa cells by means of laser scanning confocal microscopy and immunofluorescence. The presence of both CRF1 (A) and CRF2 (B) immunoreactivities was evident in the cytoplasm of HeLa cells. Omission of the primary antibody resulted in the lack of the immune signal (see also the methodical control of Fig. 6D). The scale bar (10 μm) in panel ‘A’ applies to both micrographs.

CRFR‐specific antisera were also used to detect the intracellular localization of CRF receptor isoforms in HeLa cells. The secondary antibody recognizing the primary was conjugated to Cy3 fluorophore. Methodical control was prepared by the omission of the primary antibodies. As shown by Fig. 2A,B, both CRFR immunoreactivities were distributed throughout the cytoplasm.

### Time kinetics of hUCN1‐induced activating ERK1/2 phosphorylation in HeLa cells

**Fig. 3:**
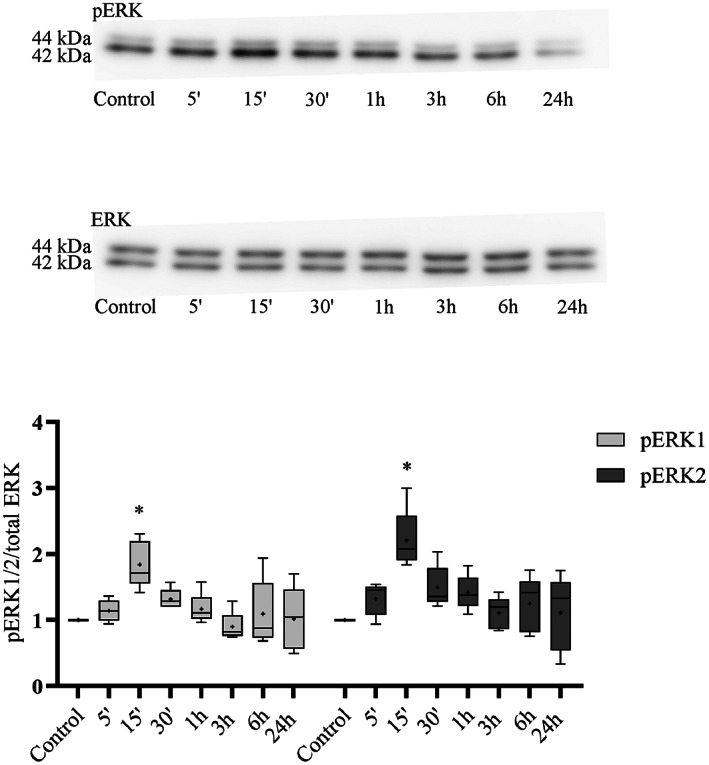
Western blot analysis of hUCN1‐induced, time‐dependent ERK1/2 phosphorylation in HeLa cells. The cells were treated with 10 nm hUCN1 for the indicated time points or were left untreated as control. A significantly stronger ERK1/2 phosphorylation (pERK1 and pERK2) was detected 15 min after the start of the treatment compared with the control group. The nonphosphorylated forms of ERK1/2 served as loading control during reprobing of the membrane. Relative ERK phosphorylation was determined by the phosphorylated/total signal ratio. The values shown are presented by box + whiskers plots, indicating median (central horizontal line), min/max values, and mean (with ‘+’ within the box) of five independently performed experiments (*n* = 5). *P < 0.05 vs. Control (two‐way ANOVA, Dunnett).

To test the possible cell proliferation‐regulating effect of hUCN1, we examined the activating phosphorylation of ERK1/2 in HeLa cells induced by the peptide. Based on our previously published findings [15] the final concentration of hUCN1 was adjusted to 10 nm. In time‐course experiments, 10 nm of hUCN1 could induce a marked, transient ERK1/2 phosphorylation peaking 15 min following the start of the treatment. After it, the signal fell back rapidly to around baseline (Fig. 3).

### Dose‐dependence of hUCN1‐induced ERK1/2 phosphorylation in HeLa cells

**Fig. 4:**
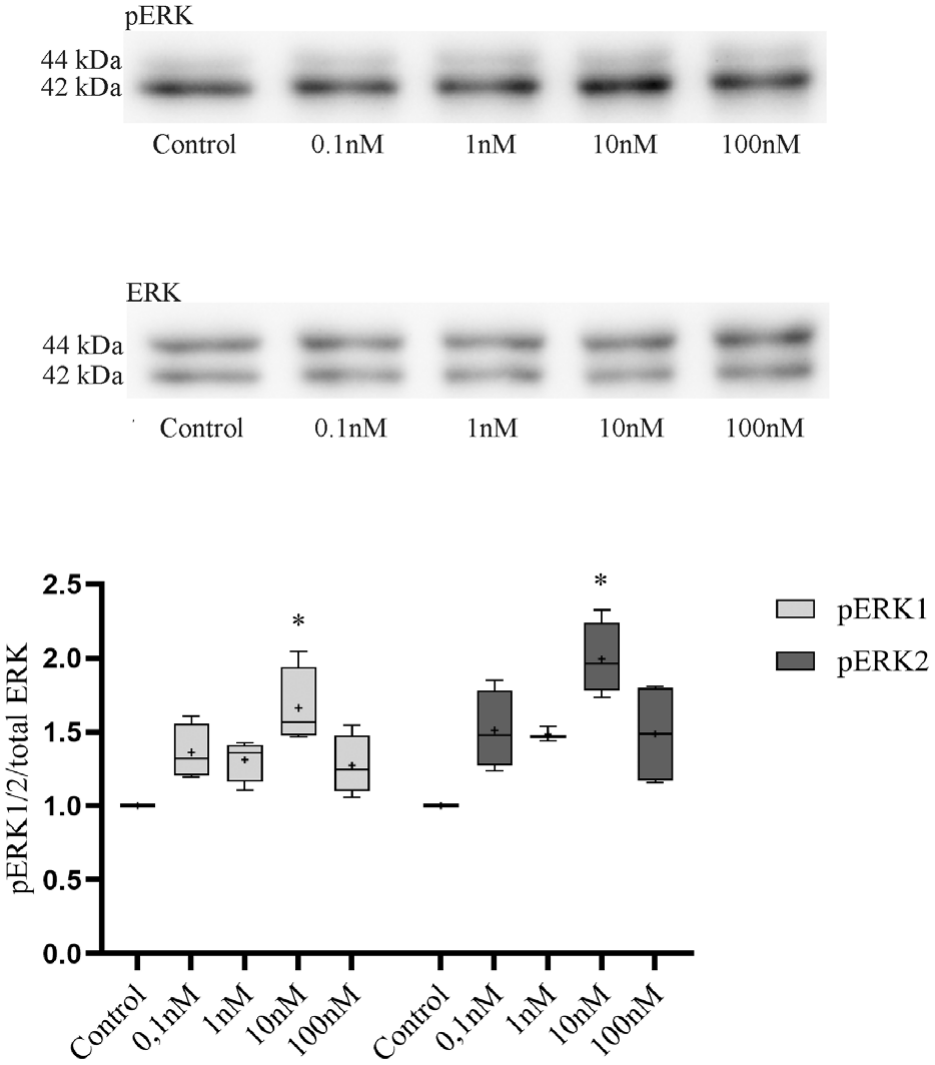
Dose‐dependence of hUCN1‐induced ERK1/2 phosphorylation by western blotting. HeLa cells were left untreated (Control) or were treated with various concentrations of hUCN1 (0.1–100 nm) for 15 min to induce the maximal phosphorylation of ERK1/2. Compared with the control group, a significant increase in ERK1/2 phosphorylation was apparent using 10 nm of hUCN1. Reprobing of the membrane for total ERK1/2 protein served the purpose of loading control. Relative protein phosphorylation was determined by the phosphorylated/total signal ratio. The values shown are presented by box + whiskers plots, indicating median (central horizontal line), min/max values, and mean (with ‘+’ within the box) of four independently performed experiments (*n* = 4). *P < 0.05 Control vs. 10 nm hUCN1 (two‐way ANOVA, Dunnett).

To see whether 10 nm of hUCN1 was indeed the most effective concentration for the treatment of HeLa cells, dose–response experiments were performed with various dilutions of hUCN1. Compared with the control group 10 nm of human urocortin1 could induce the most robust ERK1/2 phosphorylation 15 min after the start of the treatment, while other dilutions of hUCN1 proved to be less effective (Fig. 4).

### Inhibition of MEK in hUCN1‐treated HeLa cells

**Fig. 5:**
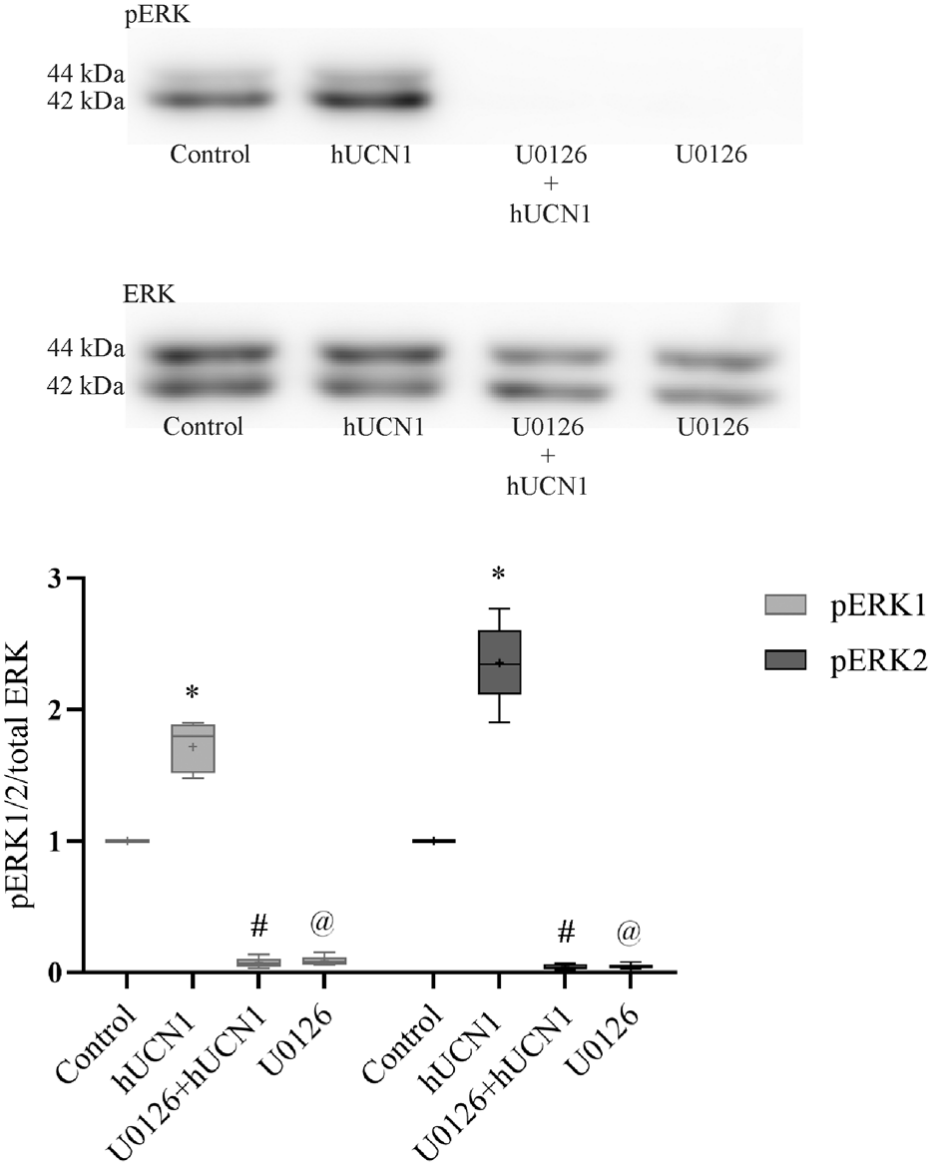
The effect of MEK inhibition on hUCN1‐induced ERK1/2 phosphorylation in HeLa cells by western blotting. HeLa cells were pretreated with the MEK inhibitor U0126 (at 20 μm final concentration), which can effectively inhibit the kinase activity of MEK. The treatment with the inhibitor started 30 min before and then continued during the last 15 min (altogether 45 min) in the simultaneous presence of 10 nm hUCN1 (to induce maximal ERK1/2 phosphorylation). The inhibitor could significantly reduce both baseline and hUCN1‐induced ERK1/2 phosphorylation. Reprobing of the membrane for total ERK1/2 served as loading control. The phosphorylated/total signal ratio was used to determine relative protein phosphorylation. The values shown are presented by box + whiskers plots, indicating median (central horizontal line), min/max values, and mean (with ‘+’ within the box) of five independently performed experiments (*n* = 5). *P < 0.05 Control vs. hUCN1 (10 nm); #*P* < 0.05 hUCN1 (10 nm) vs. U0126 + hUCN1 (10 nm); @*P* < 0.05 Control vs. U0126 (two‐way ANOVA, Tukey).

In order to examine the role of MEK (mitogen‐activated protein kinase/extracellular signal‐regulated kinase that activates ERK1/2) in hUCN1‐induced ERK1/2 phosphorylation, a MEK inhibitor chemical compound was used in Hela cells. Pretreatment of the cells with U0126 could completely abolish the ERK1/2‐activating effect of hUCN1. Compared with the control group the inhibitor alone could reduce even basal EKR1/2 phosphorylation to below detectable. Based on these results MEK appears to play a pivotal role in hUCN1‐induced ERK1/2 phosphorylation in HeLa cells (Fig. 5).

### Intracellular localization of phosphorylated ERK1/2 (pERK1/2) in hUCN1‐treated HeLa cells

**Fig. 6:**
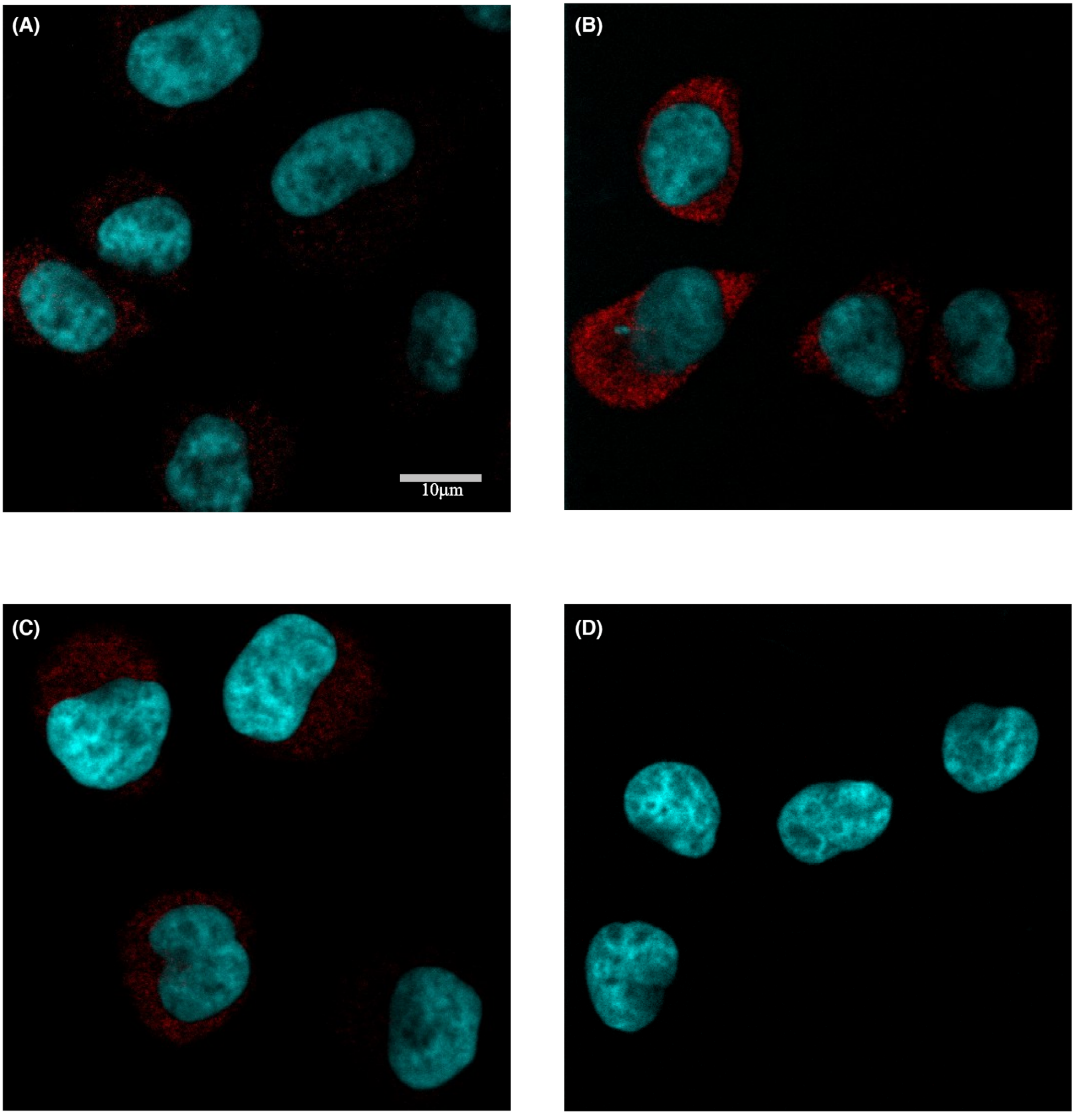
Intracellular distribution of phospho‐ERK1/2 in HeLa cells by means of immunofluorescence and laser scanning confocal microscopy. (A) barely detectable phospho‐ERK1/2 signal in the cytoplasm of untreated HeLa cells. (B) Elevated pERK1/2 signal (red) 15 min after the start of hUCN1 treatment, predominantly in the cytoplasm of HeLa cells. (C) Weaker cytoplasmic pERK1/2 signal in the presence of 10 nm hUCN1 for 24 h. (D) Methodical control prepared by the omission of the primary antibody showed no noticeable immune signal at the applied microscope settings. The scale bar (10 μm) in Panel (A) applies to all micrographs. Nuclei were counterstained using Hoechst 33342 (Calbiochem, La Jolla, CA, USA).

We also investigated the intracellular localization of pERK1/2 by immunofluorescence and laser scanning confocal microscopy. In untreated control samples, we could detect a barely noticeable phospho‐ERK1/2 signal in the cytoplasm of individual HeLa cells. Fifteen minutes after the start of 10 nm hUCN1 treatment, a stronger pERK1/2 signal was apparent compared with the control group and its localization was also cytoplasmic. By the end of the treatment day, a weaker, cytoplasmic pERK1/2 signal could still be detected matching the kinetics of the pERK1/2 signal detected on western blots (Fig. 6).

### Selective inhibition of CRF1 and CRF2

**Fig. 7:**
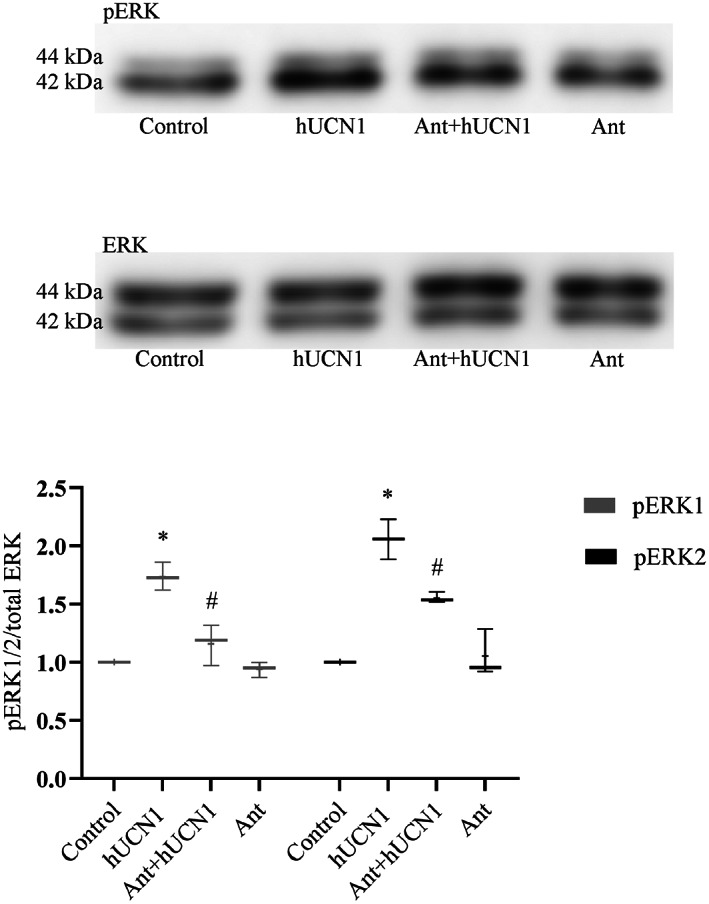
The effect of CRF1 inhibition on hUCN1‐induced ERK1/2 phosphorylation by western blotting. HeLa cells were pretreated with the CRF1‐specific antagonist Antalarmin at 100 nm final concentration. Inhibition was started 30 min before the urocortin treatment and was continued during the last 15 min (altogether 45 min) in the simultaneous presence of 10 nm hUCN1 (to induce maximal ERK1/2 phosphorylation). Compared with the hUCN1‐treated group Ant could markedly reduce hUCN1‐stimulated ERK1/2‐activating phosphorylation. Reprobing of the membrane for total ERK1/2 served as loading control. The phosphorylated/total signal ratio was used to determine relative protein phosphorylation. The values shown are presented by box + whiskers plots, indicating median (central horizontal line), min/max values, and mean (with ‘+’ within the box) of three independently performed experiments (*n* = 3). *P < 0.05 Control vs. hUCN1 (10 nm), #*P* < 0.05 hUCN1 (10 nm) vs. Ant + hUCN1 (10 nm; two‐way ANOVA, Tukey).

The involvement of CRF receptors in hUCN1‐induced ERK1/2 phosphorylation was examined by the addition of selective chemical inhibitors of CRFR isoforms. Pretreatment of the cells with the CRF1‐specific inhibitor Antalarmin (Ant) could significantly reduce the activating phosphorylation of ERK1/2 induced by 10 nm hUCN1 (Fig. 7).

**Fig. 8:**
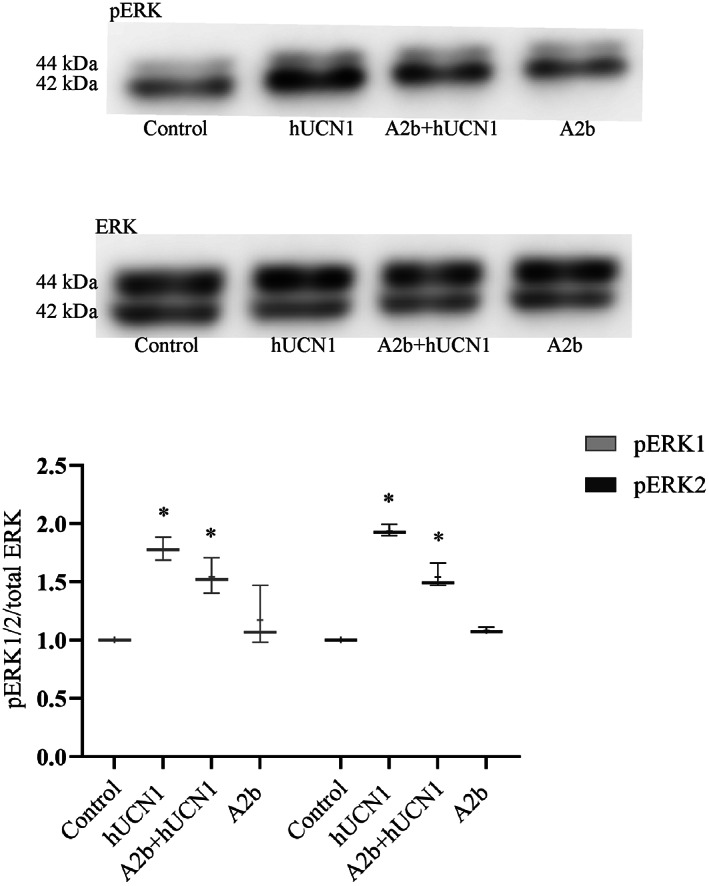
The effect of CRF2‐inhibition onto hUCN1‐induced ERK1/2 phosphorylation by western blotting. Pretreatment of cells with the CRF2‐selective antagonist A2b at 100 nm final concentration was started 30 min before the hUCN1 treatment and continued during the last 15 min (altogether 45 min) in the simultaneous presence of 10 nm hUCN1 (to induce maximal ERK1/2 phosphorylation). A2b could not markedly reduce the ERK1/2‐phosphorylating effect of 10 nm hUCN1, significantly increased ERK1/2 phosphorylation by hUCN1 was observable even in the presence of the inhibitor. Reprobing of the membrane for total ERK1/2 served as loading control. Relative protein phosphorylation was determined by the phosphorylated/total signal ratio. The values shown are presented by box + whiskers plots, indicating median (central horizontal line), min/max values, and mean (with ‘+’ within the box) of three independently performed experiments (*n* = 3). *P < 0.05 vs. Control (two‐way ANOVA, Tukey).

In contrast to the previous observation, the CRF2‐specific inhibitor Astressin 2b (A2b) was not able to reduce the ERK1/2 phosphorylating effect of hUCN1 to the degree of measurable significance (Fig. 8).

### hUCN1 stimulates the expression of E2F‐1 protein

**Fig. 9:**
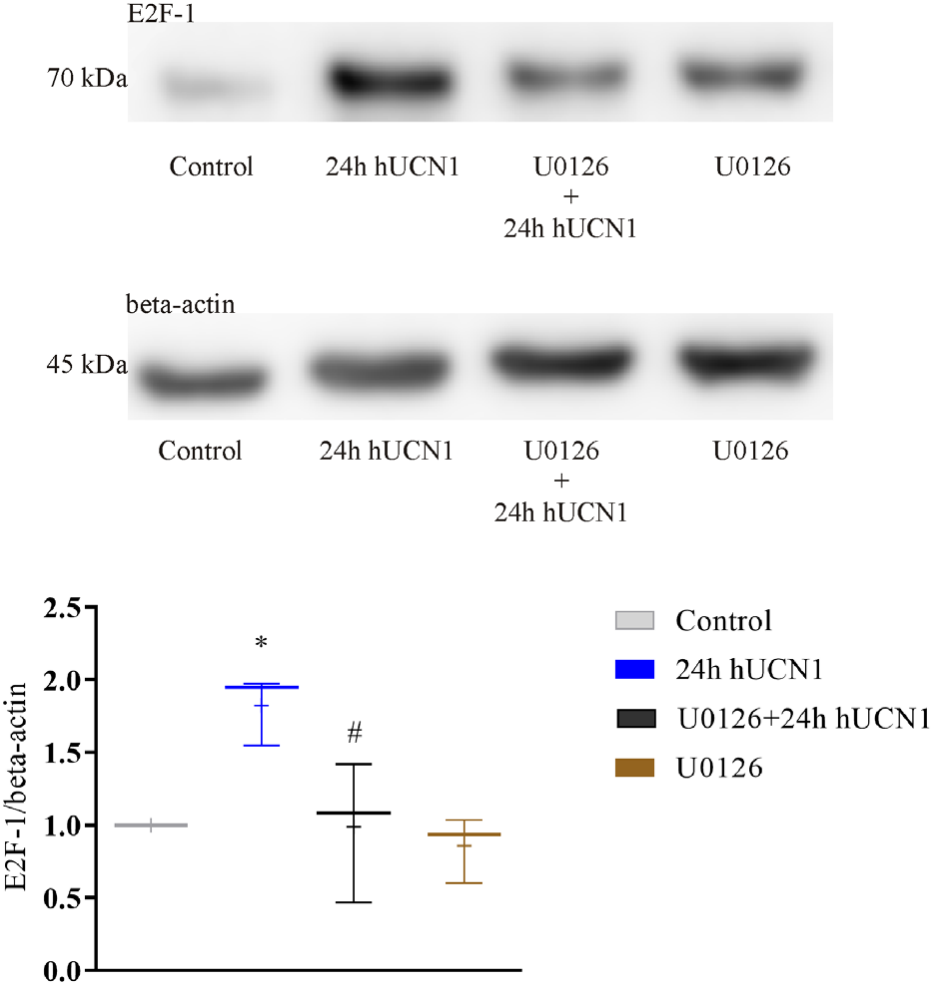
hUCN1‐induced E2F‐1 protein expression measured by western blotting. Compared with the untreated control an increased expression of E2F‐1 transcription factor was detected after 24 h in the presence of 10 nm hUCN1. This effect of hUCN1 was blocked by U0126, the selective inhibitor of MEK. Reprobing of the membranes for beta‐actin served as loading control. The values shown are presented by box + whiskers plots, indicating median (central horizontal line), min/max values, and mean (with ‘+’ within the box) of three independently performed experiments (*n* = 3). *P < 0.05 vs. Control, #*P* < 0.05 24 h hUCN1 (10 nm) vs. U0126 + 24 h hUCN1 (10 nm; one‐way ANOVA, Tukey).

Human urocortin could also increase the expression of the E2F‐1 transcription factor, a pivotal activator of S‐phase genes. Twenty‐four hours after the start of hUCN1 treatment, a marked elevation in the level of E2F‐1 could be detected by western blotting, while administration of the MEK inhibitor U0126 significantly reduced this effect of hUCN1 (Fig. 9).

### Increased PKB/Akt phosphorylation by hUCN1

**Fig. 10:**
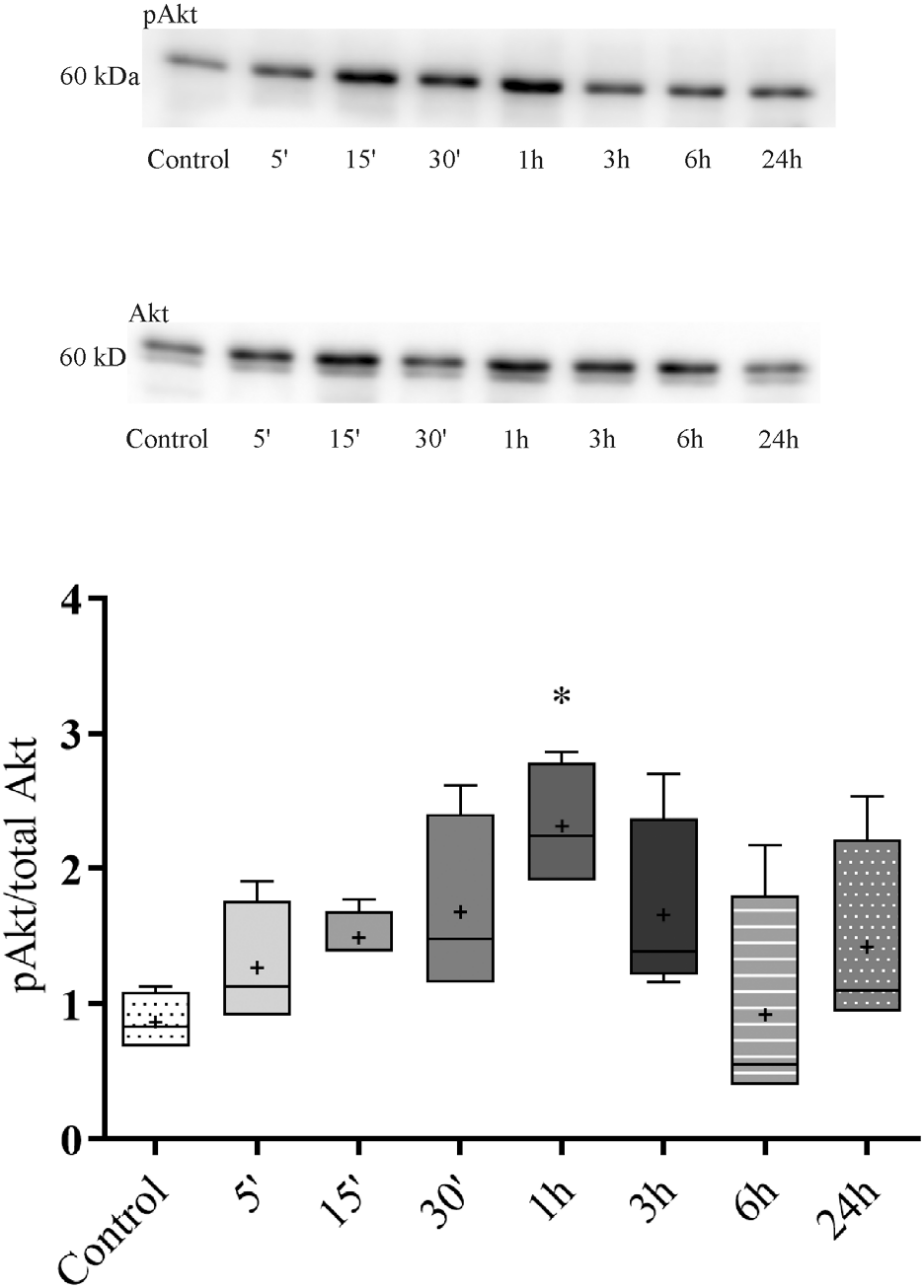
Time dependence of hUCN1‐induced PKB/Akt phosphorylation by western blotting. A marked increase in PKB/Akt phosphorylation was measured in the presence of 10 nM hUCN1 that peaked 1 h after the start of the treatment. The membrane was reprobed for total Akt, which served as loading control. Relative protein phosphorylation was determined by the phosphorylated/total signal ratio. The values shown are presented by box + whiskers plots, indicating median (central horizontal line), min/max values, and mean (with ‘+’ within the box) of four independently performed experiments (*n* = 4). *P < 0.05 vs. Control (one‐way ANOVA, Dunnett).

In time‐course experiments we also examined the activating phosphorylation of the serine/threonine‐specific enzyme PKB/Akt, a central regulator of cell survival. One hour after the start of hUCN1 treatment a significant increase in PKB/Akt phosphorylation was detected, then the signal intensity fell rapidly back to around baseline (Fig. 10).

### hUCN1 decreases the level of p53 in HeLa cells

**Fig. 11:**
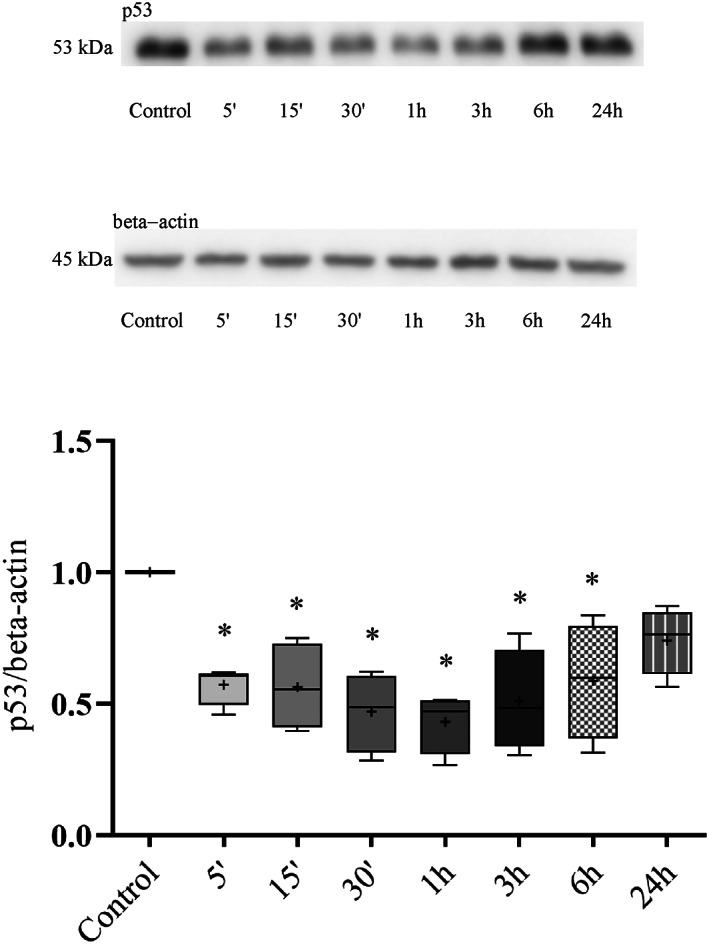
The expression of p53 protein in hUCN1‐stimulated Hela cells by western blotting. A significant decrease in p53 expression was detected in the presence of 10 nm hUCN1 for the indicated time points (5 min to 6 h), then the signal intensity rose to around baseline. The beta‐actin signal served as loading control. Relative p53 protein level was determined by the p53/beta‐actin ratio. The values shown are presented by box + whiskers plots, indicating median (central horizontal line), min/max values, and mean (with ‘+’ within the box) of four independently performed experiments (*n* = 4). *P < 0.05 vs. Control (one‐way ANOVA, Dunnett).

We examined the possible contribution of the cell cycle regulatory protein p53 in hUCN1‐stimulated signaling pathways in lysates of HeLa cells. As indicated in Fig. 11, 10 nm hUCN1 could induce a significant, sustained decrease in the level of p53 at various time points (5′–6 h), then the signal intensity returned to around baseline by the end of the treatment day (Fig. 11).

### Increased proliferation of HeLa cells measured by MTT assay

**Fig. 12:**
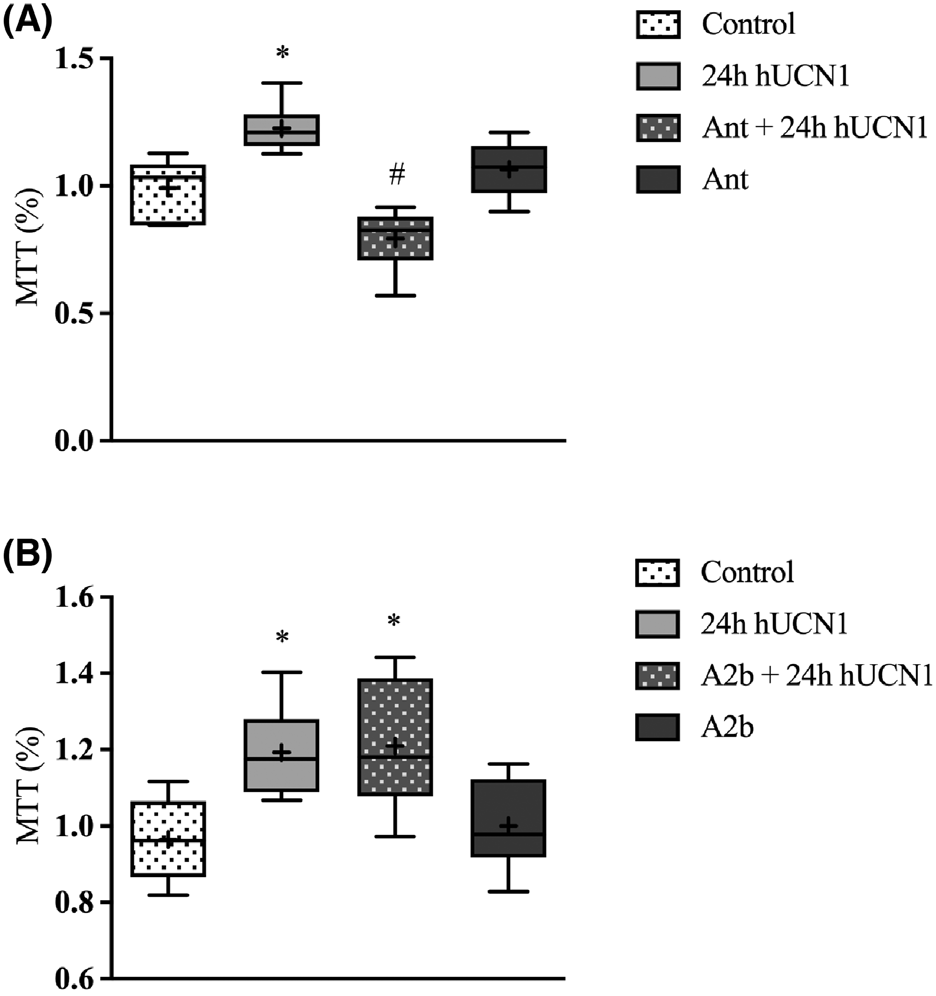
Increased MTT production of HeLa cells in the presence of 10 nm hUCN1. A significant increase in cell proliferation was measured by the applied MTT assay in the presence of 10 nm hUCN1 for 24 h compared with the untreated (Control) group. This effect of hUCN1 could be prevented in the presence of Antalarmin, a chemical inhibitor of CRF1 (Panel A). On the contrary, Astressin 2b, a specific inhibitor of CRF2 was not able to abolish the cell proliferation‐stimulating effect of hUCN1 (Panel B). The values shown are presented by box + whiskers plots, indicating median (central horizontal line), min/max values, and mean (with ‘+’ within the box) of six independently performed experiments (*n* = 6, Panel A and B). Panel A: *P < 0.05 Control vs. 24 h hUCN1 (10 nm), #*P* < 0.05 24 h hUCN1 (10 nm) vs. Ant + 24 h hUCN1 (10 nm); Panel B: *P < 0.05 vs. Control (one‐way ANOVA, Tukey).

The MTT cell viability and proliferation assay was used to examine whether hUCN1 had any effect on the proliferation of HeLa cells. The cultures were kept in their regular culturing medium as described above or in a medium supplemented with 10 nm hUCN1. In the presence of the peptide, a significant increase in MTT production was measured 24 h after the start of hUCN1 treatment when compared to values without hUCN1 treatment. This effect of 10 nm hUCN1 could be blocked using the CRF1‐specific inhibitor Antalarmin, while Astressin 2b, a chemical inhibitor of CRF2, could not prevent the hUCN1‐stimulated increase in cell proliferation (Fig. 12).

## Discussion

Urocortin members of the CRF ligand family are expressed throughout the CNS and also in peripheral organs, such as the heart, placenta, and gastrointestinal tract [16]. The effects of CRF family neuropeptides mediated through specific CRF receptors are extremely diverse, ranging from the regulation of stress responses, or anxiety, via influencing feeding behavior, to the modulation of cardiovascular and immune reactions [16, 17]. Various actions of CRF neuropeptides and their receptors can also be important regulators of tumor development (see references from [15]). Urocortin, CRF, and their receptors have been detected in various human tumors, such as in breast cancer, glioma cell lines, and others [17, 18]. According to Wang and Li [19], urocortin can inhibit the growth of tumor cells by activating CRF1.

The MAPK isoforms ERK1 and 2 are key regulators of cell proliferation both in healthy and malignant cells. CRF receptors are coupled to Gαs that can stimulate adenylate cyclase, which in turn increases intracellular cAMP production [20]. In neuronal cells the activating phosphorylation of the MAP kinase cascade through CRF receptors occurs by the stimulation of the cAMP/PKA pathway [21], while in mouse cardiomyocytes and Chinese hamster ovary cells MAPK activation is PKA‐independent [20], indicating a cell‐type‐specific process. Other proteins could also be involved in the activation of ERK1/2 MAPK, such as PKB/Akt, PKC, and Raf‐1 [22]. In human pregnant myometrial cells, urocortin stimulates the phosphorylation of ERK1/2 MAP kinase, but the activating phosphorylation can be prevented in the presence of the MEK inhibitor U0126 [23]. A similar pattern was also described in our previous work [15] in MCF7 breast cancer cells and is supported by the present study likewise. Urocortin also protects the isolated rat heart against ischaemic and reperfusion injury in a MAPK‐dependent manner [21] by activating the phosphorylation of ERK1/2 [22]. Others have reported that urocortins (1, 2, and 3) can protect the rat heart from reperfusion injury not only *in vitro* but also *in vivo* via upregulation of the MAPK EKR1/2 signaling pathway [24, 25].

MAPKs are central regulatory elements of cell division signaling. In our current experiments, hUCN1 could maximally stimulate the activating phosphorylation of the MAPK ERK1/2 at a concentration as low as 10 nm 15 min after the start of the treatment, then the signal rapidly fell back to baseline. Active, phosphorylated ERK1/2 immunoreactivity could be detected predominantly in the cytoplasm by means of fluorescence microscopy. Transient cytoplasmic ERK1/2 activation is correlated with increased cell proliferation in PC12 rat pheochromocytoma tumor cells (for references see [15]). Pretreatment of HeLa cells with the MEK inhibitor U0126 could completely abolish the ERK1/2‐phosphorylating effect of the peptide, pointing to a central role of MEK in hUCN1‐induced ERK1/2 activation of HeLa cells.

In the Ishikawa endometrial cell line, CRF1 mediates antiproliferative actions, while in peripheral blood lymphocytes, it supports apoptosis [18]. In CRF receptor‐expressing HL‐1 cardiac cells urocortin‐induced ERK1/2 phosphorylation can be inhibited either by the CRF1‐specific chemical inhibitor Antalarmin or by the specific CRF2 antagonist antisauvagine‐30 [26]. The ERK1/2‐phosphorylating effect of urocortin can also be blocked by Astressin 2b, a chemical inhibitor of CRF2, as it was experienced in rat esophageal cells [27].

Stimulation of CRF1 by its ligands CRF or UCN1 is an important initial step in stress conditions like anxiety, depression, or inflammatory diseases of the intestinal tract. Hence, the inhibition of the receptor's activity is often favorable in experimental models of these conditions, and deciphering the cellular and molecular reactions in the background is of potential medical relevance. We could detect the presence of CRF1 and 2 by western blotting and immunofluorescence microscopy in HeLa cells using commercially available CRFR isoform‐specific antibodies. In our earlier study with MCF7 human breast carcinoma cells [15], like in the work of others [10, 11] CRF1 immunoreactivity was localized mostly to and along the plasma membrane. The CRF2 immune signal, on the contrary, had a broader distribution pattern with a more granular appearance and not only along the cell surface but reaching deeper into the cytoplasm as well. In HeLa cells, at the same time, the immune signal of both CRF1 and 2 was detectable not just along the cell surface but also reaching deeper into the cytoplasm. In this cell type, the signal pattern contained bigger granules in the case of CRF1 and finer ones for CRF2. Since the culturing conditions and all steps of the methodology were identical during the fluorescence microscopic work with both cell lines (MCF7 and HeLa), the observed differences in signal distribution (CRF1 in MCF7 closer to the membrane while CRF1 in HeLa and CRF2 also deeper in the cytoplasm of both cell lines)—we attribute to cell‐type‐specific features. We have not examined the potential heterodimerization of CRF1 and 2 receptors, which can also have a serious effect on their subcellular localization [10].

The general experience in stress signaling is that CRF1 mediates mainly stress‐adaptive responses, while signals originating from CRF2 mostly dampen the effects of CRF1 receptor and that way ameliorate stress reactions of biological systems [28, 29]. Pretreatment of HeLa cells with the CRF1 inhibitor Antalarmin could significantly decrease the ERK1/2‐phosphorylating effect of hUCN1, indicating the importance of CRF1 receptor in hUCN1‐induced ERK1/2 phosphorylation in this cell type. On the contrary, we could not detect a statistically significant inhibitory effect in the presence of the CRF2 inhibitor Astressin 2b during hUCN1 treatment of HeLa cells.

Furthermore, we examined the effect of 10 nm hUCN1 treatment on the proliferation of HeLa cells by MTT assay. Our results showed a significant increase in MTT production 24 h after the start of hUCN1 treatment. The cell proliferation‐stimulating effect of hUCN1 could also be inhibited using Antalarmin, a specific inhibitor of CRF1, while pretreatment with CRF2 inhibitor Astressin 2b did not show such an effect—a striking difference compared with that seen earlier with MCF7 cells, where both receptor inhibitors could reduce MTT production [15]. The observed differences are pointing to important cell‐type‐specific deviations in urocortin's signaling mechanism of action. The converted MTT tetrazolium compound is used to determine the number of healthy, viable cells. Based on our results we can conclude that 24 h of 10 nm hUCN1 treatment increased the number of live HeLa cells, which could be inhibited by Antalarmin, indicating the significance of CRF1 in this signaling process induced by urocortin in HeLa cells.

We tried to decipher the molecular background of the observed increase in cell proliferation by measuring the expression of E2F‐1, the pivotal activator of S‐phase genes. We could detect a significant increase in the level of E2F‐1 24 h after the start of hUCN1 treatment by western blotting. Meanwhile, the MEK inhibitor U0126 could inhibit the E2F‐1‐stimulating effect of hUCN1, suggesting the involvement of MEK in these urocortin‐stimulated signaling steps.

Apoptosis is another phenomenon that is frequently mediated via CRF receptors. According to Tsatsanis *et al*. [30], urocortin can induce apoptosis in macrophages via CRF2, including the activation of central pro‐apoptotic proteins, like Bad and Bax. On the contrary, in the RM‐1 mouse prostate cancer cell line, the presence of urocortin did not support apoptosis, while CRF had the opposite effect [31]. The cardioprotective actions of urocortin2 against ischemia–reperfusion (I/R) are well known, and at the same time, it decreases apoptosis in Wistar rats [32]. CRF also suppressed apoptosis in Y79 human retinoblastoma cells by inhibiting the proteolytic cleavage and activation of procaspase 3 [33].

Based on the above data, we tried to determine the contribution of the cell cycle‐ and cell survival−/apoptosis‐regulator protein p53 to hUCN1‐stimulated signaling events in HeLa cells. Data generated by western blots showed a significant decrease in p53 expression in the presence of 10 nm hUCN1, then the signal intensity rose back to around baseline 24 h after the start of the treatment. In an earlier study [15] treatment of MCF7 cells resulted in significantly increased expression of p53. The differing effects of hUCN1 on p53 expression again underline the importance of cell‐type‐specific differences in urocortin signaling under the applied settings.

Phosphatidylinositol‐3 kinase (PI3K) activation by urocortin was reported by Chandras *et al*. [34] in human monocytic THP‐1 cells, an effect mediated by CRF2, while in human embryonic kidney (HEK) 293 cells the enzyme is activated via CRF1 [35]. UCN2 can regulate glucose metabolism by inhibiting the insulin‐induced phosphorylation of ERK1/2 and Akt in C2C12 myotubes [36], and a similarly decreased Akt phosphorylation at Ser‐473 was also described in L6 myotubes by Gao *et al*. [37].

In this direction, we could also detect the activating phosphorylation of the key cell survival‐regulator PKB/Akt 1 h after the start of hUCN1 treatment. hUCN1‐induced activation of the enzyme and the decreased p53 level may both contribute to the increased survival of HeLa cells. An additional important factor worth considering is that HeLa human cervical carcinoma cells used in the current study similar to the MCF7 human ductal breast carcinoma cell line used in our previously published work [15] are both of female origin. Furthermore, in HeLa cells of cervical cancer origin, the E6 and E7 early proteins of human papilloma viruses interfere with the normal function of key signaling and cell cycle‐controlling proteins like that of retinoblastoma and p53 [38, 39, 40, 41]. Therefore we should keep sex [42] and the presence of potent signal‐distorting components of viral origin as important possible factors in mind when interpreting our data and their potential pathological and therapeutic relevance.

## Conclusions

Our results showed the presence of both CRF1 and 2 immunoreactivities in the cytoplasm of HeLa cells using isoform‐specific antisera and laser scanning confocal microscopy. We could detect transient ERK1/2 phosphorylation peaking at 15 min in the presence of 10 nm hUCN1. This effect of hUCN1 could be prevented by the MEK inhibitor U0126 suggesting the pivotal role of the MAPK kinase in hUCN1‐induced ERK1/2 activation. We also demonstrated that the ERK1/2 phosphorylating effect of 10 nm hUCN1 was initiated via CRF1, while the participation of CRF2 in this process is not confirmed by our data. hUCN1 treatment increased the expression of the E2F‐1 transcription factor, a key regulator of S‐phase genes supporting cell proliferation. This phenomenon could be blocked by U0126, the chemical inhibitor of MEK emphasizing the importance of the enzyme in hUCN1‐stimulated signaling pathways. At the same time, urocortin increased the overall production of MTT formazan crystals in HeLa cells, which could be inhibited by the CRF1‐specific inhibitor Antalarmin, but not by Astressin 2b the inhibitor of the other main CRF receptor isoform. Interestingly, in MCF7 cells [15] both receptor isoform‐specific inhibitors could reduce MTT production to a significant degree. The elevated expression of E2F‐1 and the increased production of formazan dye in the presence of urocortin are both suggestive of increased HeLa cell proliferation. While the activating phosphorylation of PKB/Akt and the decreased expression of p53 upon hUCN1 treatment support increased cell survival in the same cell type. In view of our data, we can conclude that the effect of hUCN1 in HeLa cells is mediated via CRF1 and MEK towards ERK1/2 while MEK is also critical for the induction of increased E2F‐1 expression, supporting increased cell proliferation. We believe that our results can help a better understanding of how urocortins exert their cell proliferation‐regulating effects in a cell‐type‐specific manner and it also sheds light onto their potential role in cancer formation. The above‐revealed cell‐type‐specific aspects of urocortins' signaling may also have therapeutic relevance to targeted treatments of various malignancies in the future.

## Conflict of interest

The authors declare no conflict of interest.

## Author contributions

BB performed experiments (cell culturing and treatments, investigation using western blots, MTT assay, confocal microscopy), analyzed and visualized data, and wrote the original manuscript; GB conducted confocal imaging; MV performed experiments (cell culturing and treatments, investigation using western blots, visualization); OT and AS‐H reviewed the manuscript; GS Jr engaged in conceptualization, supervision, project administration, funding acquisition, and investigation using confocal microscopy, and also reviewed and edited the text.

## Data Availability

All data are presented in the manuscript.
